# Supplementary material for: Molecular definition of group 1 innate lymphoid cells in the mouse uterus
Source: Nat Commun. 2018 Oct 29;9:4492. doi: 10.1038/s41467-018-06918-3 (PMC6206068; doi:10.1038/s41467-018-06918-3)
Supplement: Supplementary file 1 — Supplementary Information [file 41467_2018_6918_MOESM1_ESM.pdf]

Supplementary Information

Molecular definition of group 1 innate lymphoid cells in the mouse uterus

Filipovic et al.

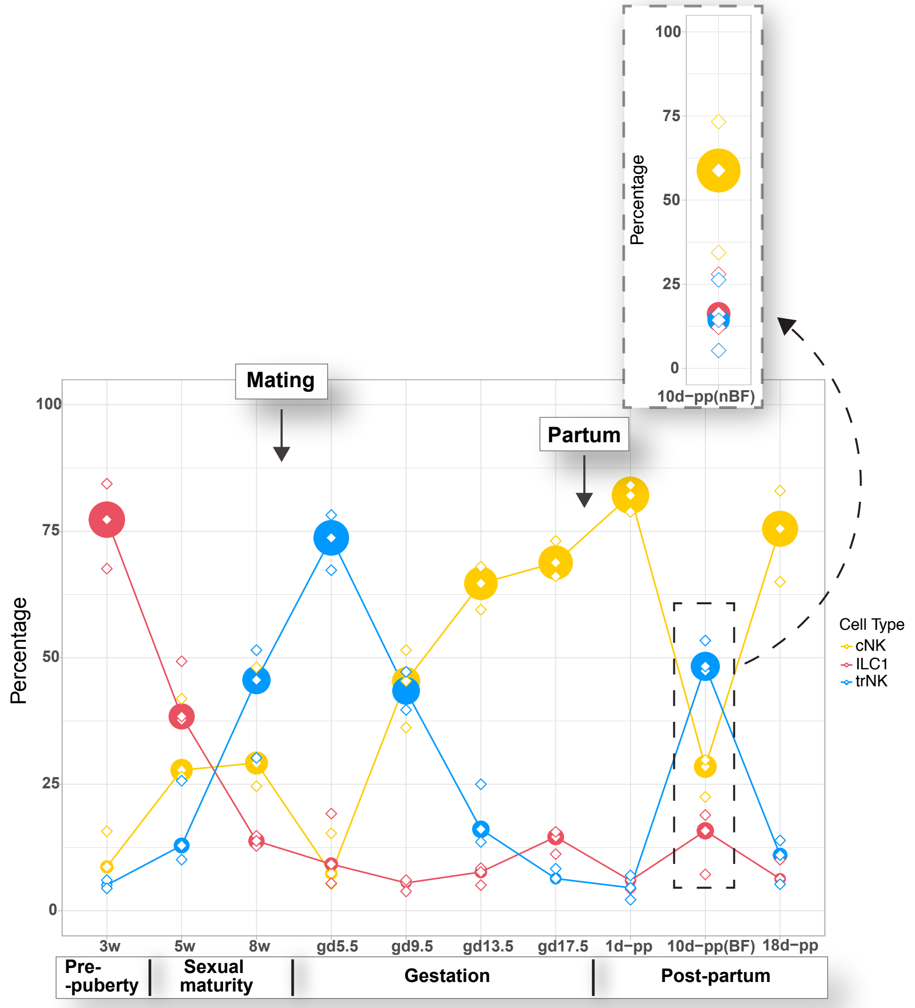

Supplementary Figure 1. Dynamic distribution of uterine g1 ILCs during reproductive life. Shown are individual data points (empty rhomboids of matching colour) of the data shown in Figure 1. Means are indicated by filled circles. w, week; gd, gestation day; pp, post-partum, BF, breast-feeding; nBF, non-breast-feeding.

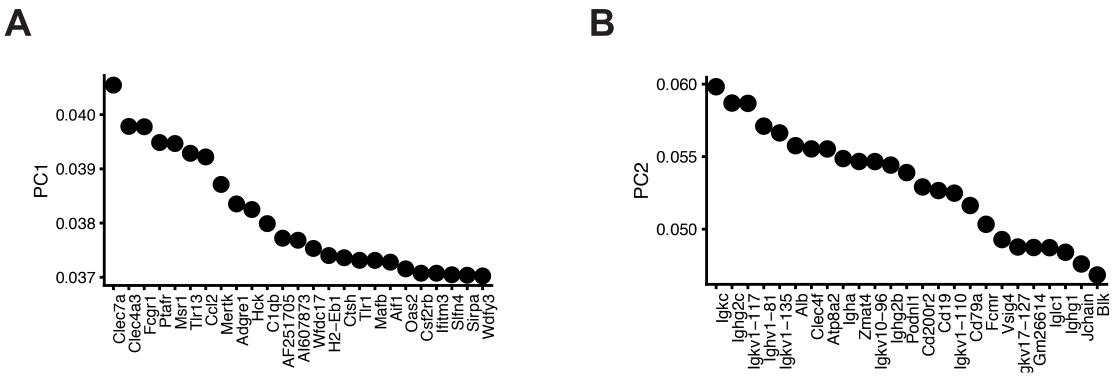

Supplementary Figure 2. First two principal components explain differences between the subsets of g1 ILCs. (**A**) Top genes explaining variance within principal component 1 (PC1). (**B**) Top genes explaining variance within principal component 2 (PC2).

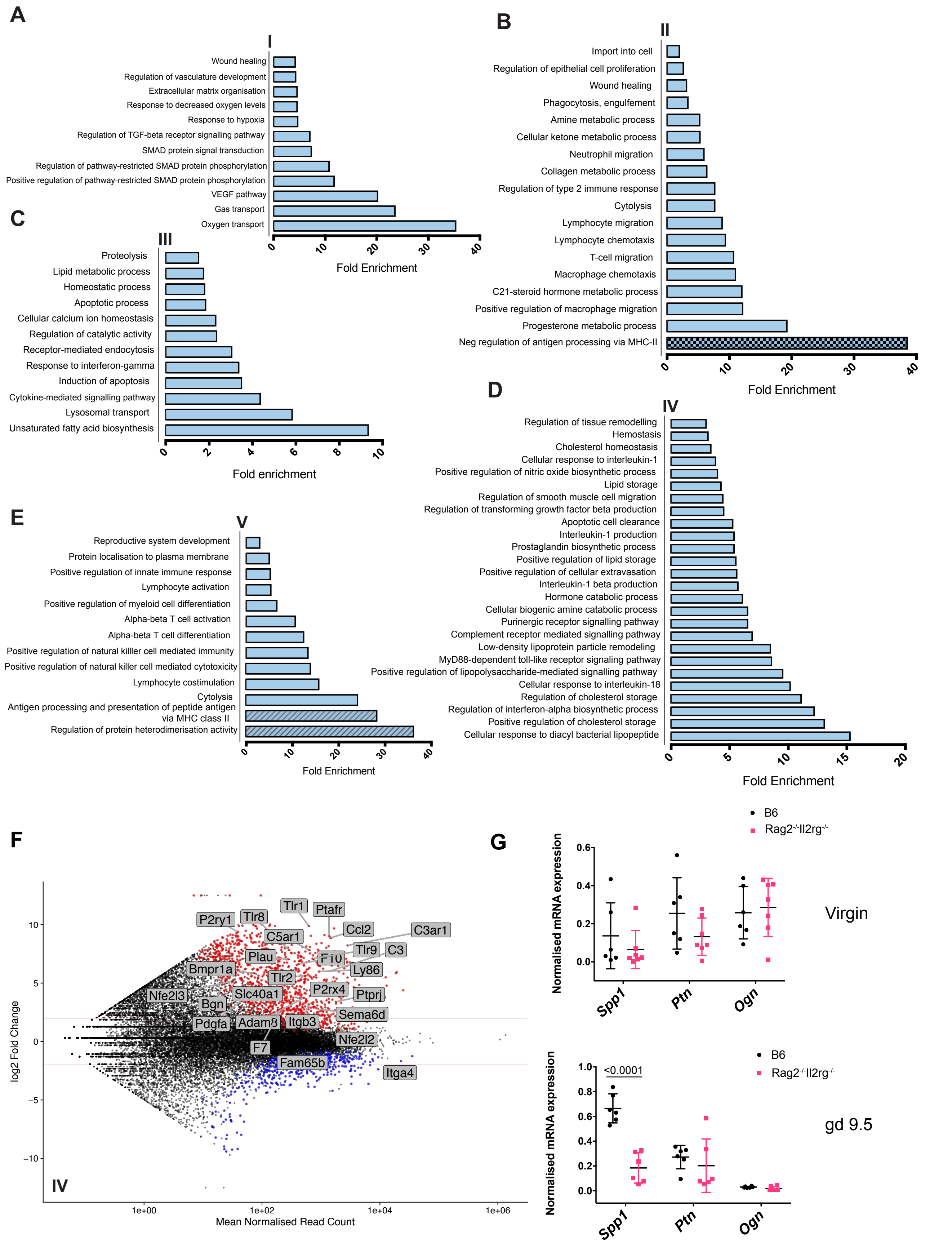

Supplementary Figure 3. Core enriched pathways and genes in uterine g1 ILCs. (**A-E**) Gene Ontology (GO) Analysis of selected comparisons for classifications by biological process showing selected pathways with fold enrichment: (**A**) GO for comparison I. (**B**) GO for comparison II. Shaded bars indicate enriched pathways in liver ILC1s. (**C**) GO-Slim for comparison III. (**D**) GO for comparison IV. (**E**) GO for comparison V. Shaded bars indicate enriched pathways in uterine ILC1s. (**F**) MA plot showing additional differentially expressed genes for comparison IV (uterine trNK vs cNK) to complement Figures 3E and Supplementary Fig. 3D. (**G**) mRNA expression levels detected by RT-qPCR for Spp1, Ptn and Ogn in whole uterine tissue from either non-pregnant (virgin) B6 and Rag2$^{-/-}$Il2rg$^{-/-}$ mice or from B6 and Rag2$^{-/-}$Il2rg$^{-/-}$ dams at mid-gestation (gd 9.5). Data are representative of five independent experiments (four technical replicates were used for each biological replicate). Statistical significance was evaluated by two-way ANOVA with multiple comparisons correction. Error bars represent mean±SD.

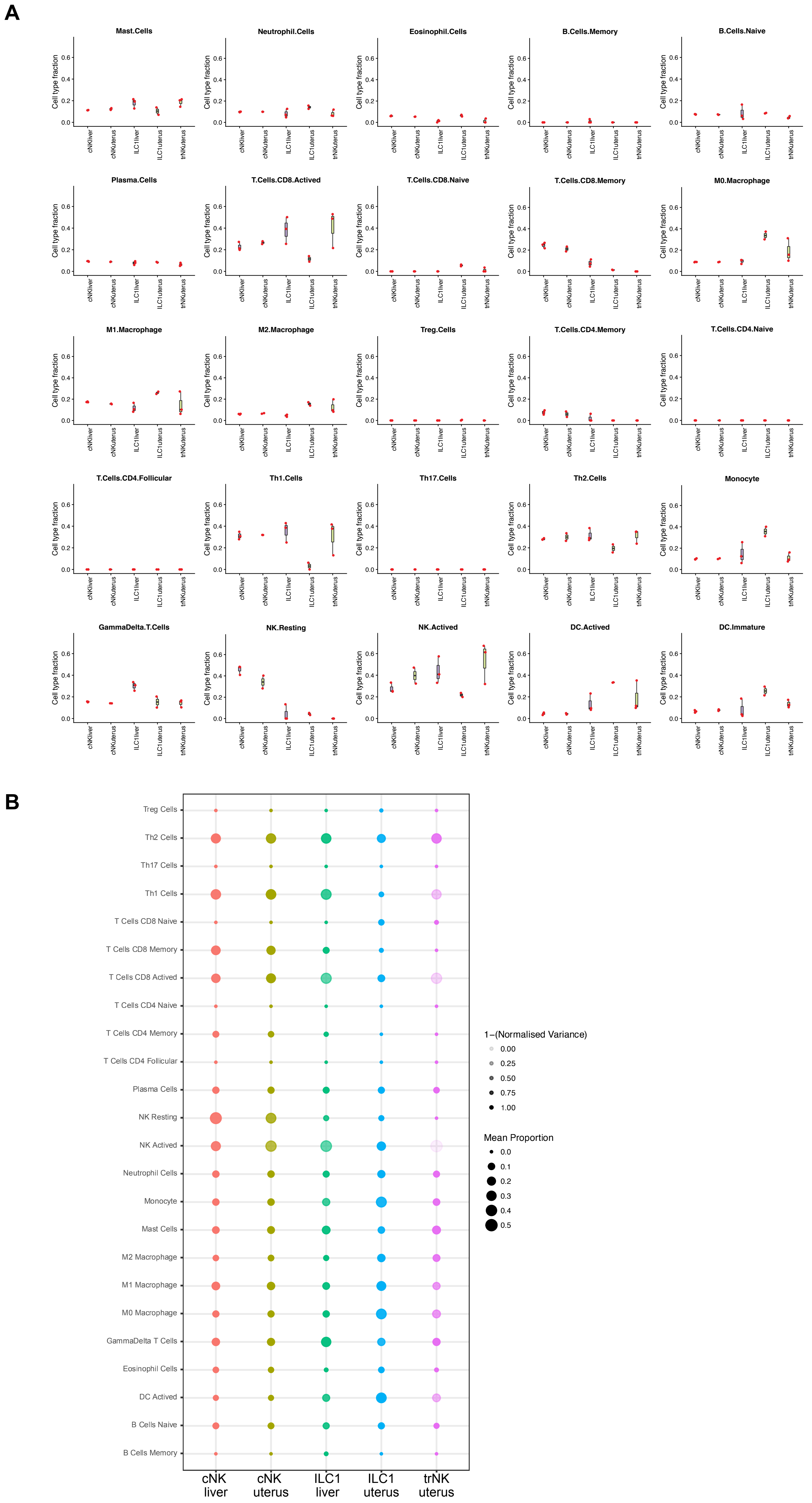

Supplementary Figure 4. A prediction model confirms unique features of uterine ILC1s. (**A**) DeconRNASeq was applied to RNA-seq data generated in this study as a deconvolution method to quantitatively estimate the relative fractions of various immune cell types within gd 9.5 g1 ILCs in the liver and uterus. (**B**) Summary of the data presented in Supplementary Figure 4A for each of the two subsets in liver and three subsets in the uterus.

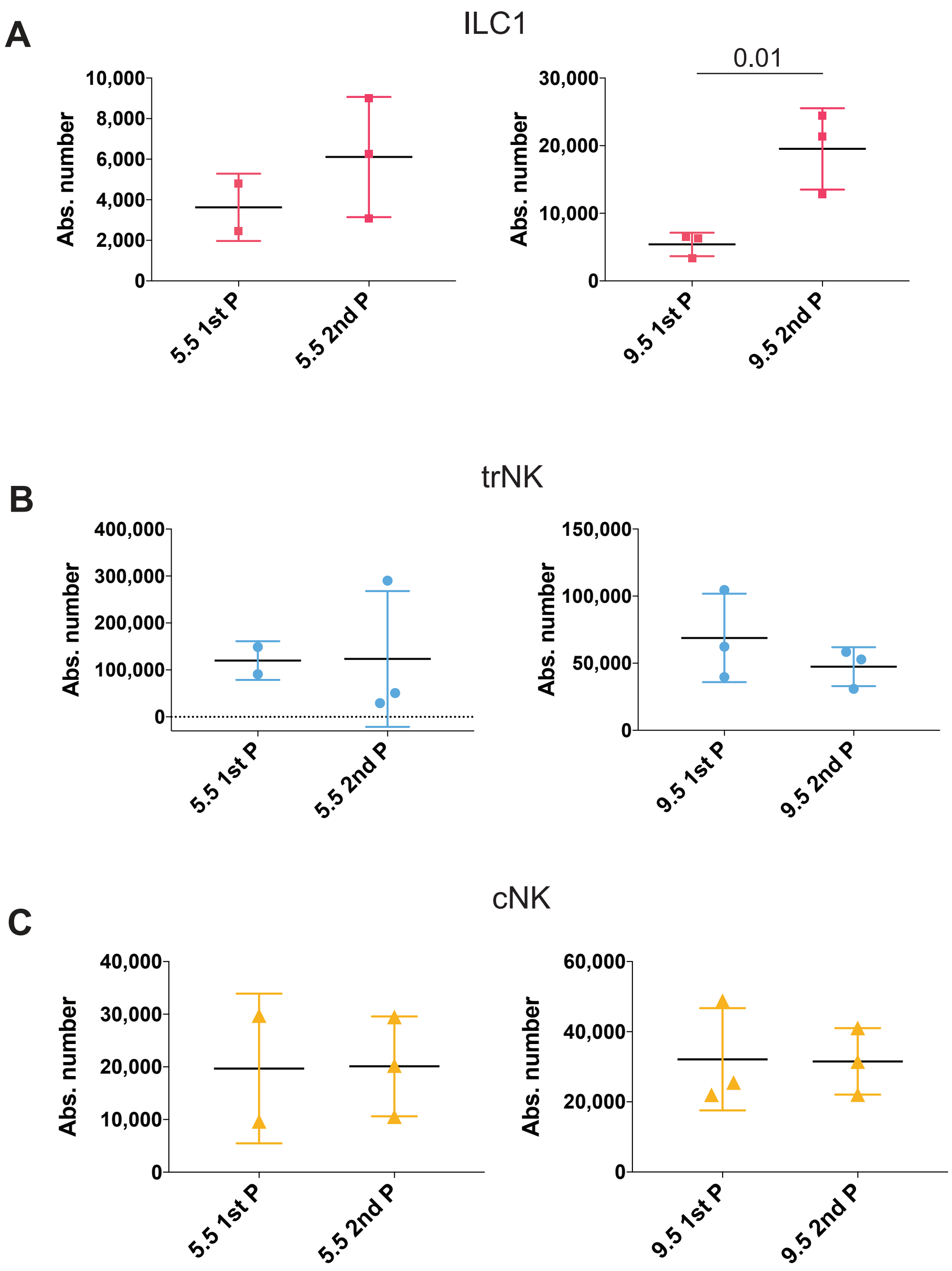

Supplementary Figure 5. Increased numbers of Eomes$^-$CD49a$^+$CXCR6$^+$ g1 ILCs specifically in second gestation. The same data presented as percentages in Figure 5 are presented here as absolute cell numbers of (**A**) ILC1s, (**B**) trNK cells, (**C**) cNK cells at gd 5.5 and gd 9.5 in first (1st P) and second (2nd P) pregnancy. Data representative of 2 or 3 independent samples and three independent experiments. Statistical significance was evaluated by two-way ANOVA with multiple comparisons correction. Error bars represent mean±SD.
