# Supplementary material for: Molecular definition of group 1 innate lymphoid cells in the mouse uterus
Source: Nat Commun. 2018 Oct 29;9:4492. doi: 10.1038/s41467-018-06918-3 (PMC6206068; doi:10.1038/s41467-018-06918-3)
Supplement: Supplementary file 3 — Description of Additional Supplementary Files [file 41467_2018_6918_MOESM3_ESM.pdf]

The legends for Supplementary Data:

Supplementary Data 1. All differentially expressed genes and pathways highlighted by the five comparisons in Figure 3A. For each of the comparisons, the first tab represents the list of differentially expressed gene (DEG I) and the tab next to it represents the gene ontology (GO) analysis for the corresponding list (i.e., GO I).
